# Isolation of 4,5-*O*-Dicaffeoylquinic Acid as a Pigmentation Inhibitor Occurring in* Artemisia capillaris* Thunberg and Its Validation* In Vivo*

**DOI:** 10.1155/2016/7823541

**Published:** 2016-07-26

**Authors:** Nadia Tabassum, Ji-Hyung Lee, Soon-Ho Yim, Galzad Javzan Batkhuu, Da-Woon Jung, Darren R. Williams

**Affiliations:** ^1^New Drug Targets Laboratory, School of Life Sciences, Gwangju Institute of Science and Technology, Gwangju 500-712, Republic of Korea; ^2^Department of Pharmaceutical Engineering, Dongshin University, 185 Geonjaero, Naju, Jeonnam 520-714, Republic of Korea; ^3^School of Engineering and Applied Sciences, National University of Mongolia, 14200 Ulaanbaatar, Mongolia

## Abstract

There is a continual need to develop novel and effective melanogenesis inhibitors for the prevention of hyperpigmentation disorders. The plant* Artemisia capillaris* Thunberg (Oriental Wormwood) was screened for antipigmentation activity using murine cultured cells (B16-F10 malignant melanocytes). Activity-based fractionation using HPLC and NMR analyses identified the compound 4,5-*O*-dicaffeoylquinic acid as an active component in this plant. 4,5-*O*-Dicaffeoylquinic acid significantly reduced melanin synthesis and tyrosinase activity in a dose-dependent manner in the melanocytes. In addition, 4,5-*O*-dicaffeoylquinic acid treatment reduced the expression of tyrosinase-related protein-1. Significantly, we could validate the antipigmentation activity of this compound* in vivo*, using a zebrafish model. Moreover, 4,5-*O*-dicaffeoylquinic acid did not show toxicity in this animal model. Our discovery of 4,5-*O*-dicaffeoylquinic acid as an inhibitor of pigmentation that is active* in vivo* shows that this compound can be developed as an active component for formulations to treat pigmentation disorders.

## 1. Introduction

The visible color of the mammalian skin, hair, and eyes results from the quantity, quality, and epidermal distribution of the melanosomes. These are organelles produced by specialized dendritic cells, called melanocytes [1]. Melanin pigment is synthesized in the melanosomes, which is transported to keratinocytes by melanocyte dendrites. Thus, the distribution pattern of melanin determines skin color [2]. Melanin provides broad wavelength protection from solar UV radiation and absorbs free radicals generated in the skin [3]. Melanogenesis is regulated by the expression of enzymes involved in melanin formation. More than 100 proteins are involved in regulating pigmentation [2, 4]. For example, melanogenesis is initiated with tyrosine oxidation to dopaquinone, which is catalyzed by the key regulatory enzyme, tyrosinase. Dopaquinone is further converted to eumelanin by intramolecular cyclization and polymerizations reactions [5]. Dysregulation in melanogenesis may result in the accumulation of excessive levels of pigmentation, producing disorders such as melasma, age spots, and sites of solar keratosis. Changes in skin color are also desired for cosmetic reasons, which has produced a significant global market for skin lightening products [6]. Lightening products and therapeutics include hydroquinones, retinoids, and tyrosinase inhibitors. However, these treatments may cause problems, including mutations, toxicity, and ochronosis (blue-black hyperpigmentation of skin) [7].

In this study, we used* in vitro* melanocyte-based screening to screen extracts from the plant* Artemisia capillaris* Thunberg (*A. capillaris*; Oriental Wormwood) to discover novel melanin regulatory compounds.* A. capillaris* has been traditionally used as a herbal medicine in Korea and China since ancient times [8]. Extracts/preparations from this plant exhibit various pharmacological activities, such as antiviral infection [9], antioxidant effects [10], hepatoprotective properties [11], and anti-inflammatory effects [11, 12]. Numerous active compounds have been extracted from* A. capillaris*, such as phenolic compounds, flavonoids, flavonoid glycosides, and coumarins [11, 13]. We isolated an antipigmentation compound from the methanol extract of* A. capillaris*, which was identified as 4,5-*O*-dicaffeoylquinic acid (4,5-diCQA). 4,5-diCQA was shown to be a pigmentation inhibitor in both melanocytes and the zebrafish vertebrate model. Significantly, no toxicity was observed in our analysis, indicating that 4,5-diCQA is an attractive candidate for further development as a pharmaceutical or cosmetic depigmenting agent.

## 2. Material and Methods

### 2.1. Chemicals

PTU, NaOH, DMSO (dimethyl sulfoxide), L-DOPA (-3,4-dihydroxyphenylalanine), CellLytic*™* buffer, mushroom tyrosinase, MTT (3-(4,5-dimethylthiazol-2-yl)-2,5-diphenyltetrazolium bromide), and tricaine methanesulfonate solution were purchased from Sigma (St Louis, MO, USA). L-tyrosine was purchased from Duchefa Biochemie (Haarlem, Netherland). All test compounds were dissolved in DMSO and protected from light at −20°C until use. HPLC grade solvents, acetonitrile, and methanol were obtained from Merck (Darmstadt, Germany).

### 2.2. Plant Material

The leaves and stems of* Artemisia capillaris* Thunberg were provided by Professor Soon-Ho Yim, Dongshin University, Naju, Republic of Korea.

### 2.3. Cell Culture

Murine melanoma B16-F10 cells were obtained from the American Type Culture Collection (ATCC, Manassas, VA) and cultured in DMEM supplemented with 10% FBS, 1% penicillin-streptomycin mixture (Gibco, USA). Cultured cells were maintained in a 37°C humidified incubator with 5% CO_2_.

### 2.4. Determination of Melanin Content in B16-F10 Melanocytes

Melanocytes were rinsed with phosphate buffered saline (PBS) and lysed with CellLytic buffer at 4°C. Cell extracts were spun at 13,000 rpm for 10 min at 4°C. The remaining pellet was assayed for melanin by rinsing twice with ethanol : ether (1 : 1) and dissolving in 200 *µ*L of 1 N NaOH in 10% DMSO at 80°C. A 100 *µ*L aliquot of the resulting solution was then measured for absorbance at 400 nm using microplate reader (VersaMax*™*; Molecular Devices Corporation, California, USA) [14].

### 2.5. Determination of* In Vitro* Tyrosinase Activity

Tyrosinase activity was determined as described previously [15]. Briefly, B16-F10 melanocytes were seeded in a 6-well plate at a density of 2 × 10^5^ cells/well. The melanocytes were treated with compound for 48 hr. Melanocytes were lysed using lysis buffer and centrifuged at 13,000 rpm for 10 min. 100 *µ*L of each lysate containing an equal amount of protein (250 *µ*g) was placed into a 96-well plate, and 100 *µ*L of 5 mM L-DOPA was added to each well. After incubation at 37°C for 60 min, dopachrome formation was measured at 475 nm using a microplate reader.

### 2.6. HPLC-Based Activity Profiling of* Artemisia capillaris* Extract

The dried powder of the leaves and stems from* Artemisia capillaris* was extracted with 100% MeOH and concentrated* in vacuo* to yield a MeOH extract (6 g). HPLC was performed on an Agilent HP1100 series, comprised of a degasser, a binary mixing pump, a column oven, and a DAD detector, using YMC-PAC Pro C18 (10 mm, 250 mm, 5 *µ*m) columns, in conjunction with a gradient system of MeCN and H_2_O containing 0.1% HCOOH. For activity profiling, a portion (6 g) of the MeOH extract was fractionated by semipreparative HPLC (Agilent 1100 Series, USA) using the gradient eluent system with acetonitrile (MeCN) and water containing 0.1% formic acid, that is, 20% MeCN to 60% MeCN for 50 min. The mobile phase was delivered at the flow rate of 6.0 mL/min and detection of eluate was carried out at 280 nm. A total of 23 fractions were collected, concentrated, and their biological activities were evaluated. Fraction ACMF09 was selected on the basis of its inhibition of melanogenesis to obtain potentially bioactive compounds. The ACMF09 fraction of MeOH extract of* A. capillaris* was separated on a RP-18 column with a gradient of H_2_O-MeOH started at 60 : 40 (v : v) and was kept constant for 50 min. The gradient system was then decreased to 0 : 100 and was kept constant for 20 min to yield a purified compound (7.2 mg).

### 2.7. 4,5-*O*-Dicaffeoylquinic Acid

^1^H-NMR (in CD_3_OD, 600 MHz) *δ* 7.59 (1H, d, *J* = 15.9 Hz, H-7′ or H-7′′), 7.51 (1H, d, *J* = 15.9 Hz, H-7′ or H-7′′), 7.02 (1H, d, *J* = 1.8 Hz, H-2′ or H-2′′), 7.00 (1H, d, *J* = 1.8 Hz, H-2′ or H-2′′), 6.92 (1H, dd, *J* = 8.1, 1.8 Hz, H-6′ or H-6′′), 6.90 (1H, dd, *J* = 8.1, 1.8 Hz, H-6′ or H-6′′), 6.75 (1H, d, *J* = 8.1 Hz, H-5′ or H-5′′), 6.74 (1H, d, *J* = 8.1 Hz, H-5′ or H-5′′), 6.28 (1H, d, *J* = 15.9 Hz, H-8′ or H-8′′), 6.19 (1H, d, *J* = 15.9 Hz, H-8′ or H-8′′), 5.62 (1H, br s, H-5), 5.11 (1H, br s, H-4), 4.37 (1H, br s, H-3), 2.40 (2H, H-6), 1.99 (2H, m, H-2); ^13^C-NMR (in CD_3_OD, 125 MHz) *δ* 168.7 (C-9′ or 9′′), 168.4 (C-9′ or 9′′), 149.8 (C-4′ or 4′′), 147.8 (C-7′ or 7′′), 147.7 (C-7′ or 7′′), 146.9 (C-3′ or 3′′), 146.8 (C-3′ or 3′′), 127.8 (C-1′ or 1′′), 127.7 (C-1′ or 1′′), 123.3 (C-6′ or 6′′), 116.6 (C-5′ or 5′′), 115.3 (C-2′ or 2′′), 115.2 (C-2′ or 2′′), 114.8 (C-8′ or 8′′), 117.7 (C-8′ or 8′′), 75.8 (C-1), 75.7 (C-2), 69.8 (C-3), 68.6 (C-5), 39.7 (C-6), 38.5 (C-14); ESI-MS* m/z* 515.1 [M–H]^−^ (C_25_H_24_O_12_).

### 2.8. Mushroom Tyrosinase Assay

The effect of the samples on mushroom tyrosinase activity was investigated according to the method of Zhang et al. [16] with minor modifications. In brief, mushroom tyrosinase enzyme was dissolved in 50 mM potassium phosphate buffer (pH 6.5) at a concentration of 500 units/mL. 550 *μ*L of 50 mM potassium phosphate and 50 *μ*L of tyrosinase solution were mixed with an appropriate volume of test samples in a microfuge tube and incubated for 5 min at room temperature. 100 *μ*L of 1.5 mM L-tyrosine was added to the solution and loaded into a 96-well plate. The amount of dopachrome formed in the reaction mixture was determined by measuring the absorbance at 490 nm using a microplate reader.

### 2.9. Quantitative Real-Time PCR Analysis

B16-F10 melanocytes were treated with test samples for 48 hr. Total RNA was extracted using the TRI-Solution*™* according to the manufacturer's instructions and quantified using a NanoDrop 2000 spectrophotometer (NanoDrop Technologies). cDNA synthesis was carried out from 1 *µ*g RNA using AccuPower® PCR PreMix (Bioneer) following the manufacturer's recommendation. mRNA expressions of the MITF gene, tyrosinase gene, and TRP-1 were quantified using a Power SYBT® Green PCR Master Mix (Applied Biosystems). mRNA levels were normalized with *β*-actin and fold change of expression was calculated with the ΔΔCT method. The primer sequences were as follows: mouse tyrosinase forward 5′-TACTTGGAACAAGCCAGTCGTATC-3′, reverse 5′-ATAGCCTACTGCTAAGCC CAGAGA-3′; mouse TRP-1 forward 5′- AAACCCATTTGTCTCCCAA -TGA-3′, reverse 5′-CGTTTTCCAACGG -GAAGGT A-3′, mouse MITF forward 5′-GGACTTTCCCTTATCCCATCCA-3′, reverse 5′-GCCGAGGTTGTTGGTAAAG -GT-3′. The PCR conditions were 95°C for 2 min followed by 40 cycles of 95°C for 30 s, 60°C for 1 min, and 72°C for 1 min followed by a final 30 sec extension at 72°C. Data were analyzed using the Stepone*™* software v2.3 (Applied Biosystems).

### 2.10. Origin and Maintenance of Parental Zebrafish

Adult zebrafish were obtained from a commercial dealer and 10–15 fishes were kept in 5 L acrylic tanks under the following conditions: 28.5°C, with a 14/10 hr light/dark cycle. Zebrafish were fed two times a day, 7 d/week, with live brine shrimps (*Artemia salina*). Embryos were obtained from natural spawning that was induced at the morning around 9:30 AM by turning on the light. Collection of the embryos was completed within 30 min.

### 2.11. Phenotype-Based Evaluation of Test Compounds Using Zebrafish

Zebrafish embryos were maintained in 100 mm^2^ petri dishes in embryo media at a density of 70–80 embryos per dish. Synchronized embryos were collected and arrayed by pipette at three embryos per well, in 96-well plates containing 200 *µ*L embryo medium (5 mM NaCl, 0.17 mM KCl, 0.33 mM CaCl_2_·2H_2_O, 0.33 mM MgSO_4_·7H_2_O; to provide a 60x stock solution). Test extracts were dissolved in 0.1% DMSO and added to the embryo medium from 9 to 72 hpf (63 hr exposure). Occasional stirring and replacement of the medium were done every 24 hr to ensure even distribution of the test compounds. In all experiments, 75 *µ*M PTU was used to generate transparent zebrafish without interfering with developmental process [17]. Phenotype-based evaluations of body pigmentation were carried out at 72 hpf. Embryos were dechorionated using forceps, anesthetized with tricaine methanesulfonate solution, and mounted in 3% methyl cellulose. The effects on the pigmentation of zebrafish were observed using stereomicroscopy (LEICA DFC425 C). Melanocyte area was calculated using the Image J program (National Institutes of Health, USA), as previously described [18].

### 2.12. Melanin Content and Tyrosinase Activity Determination in Zebrafish

Tyrosinase activity and melanin content was determined as described previously [19]. About 40 zebrafish embryos were treated with melanogenic modulators from 9 to 48 hpf, and sonicated in CellLytic buffer. Optical density of the supernatant was measured at 400 nm to measure melanin level. To determine tyrosinase activity, 250 *µ*g of total protein in 100 *µ*L of lysis buffer was transferred into a 96-well plate, and 100 *µ*L of 5 mM L-3,4-dihydroxyphenylalanine (L-DOPA) was added. Control wells contained 100 *µ*L lysis buffer and 100 *µ*L 5 mM L-DOPA. After incubation for 60 min at 37°C, absorbance was measured at 475 nm using a microplate reader. The blank was removed from each absorbance value, and the final activity was expressed as a percentage of the water control. PTU-treated embryos were used as a positive control.

### 2.13. Melanocyte Counting Assay

Embryos were assessed for melanocyte cell number as previously [20]. Embryos were first exposed to light to contract the melanin within the melanocytes, followed by imaging using stereomicroscopy. Melanocytes were counted within a defined head region in the micrographs.

### 2.14. Measurement of Embryo Heart Rate

The heart rate of both the atrium and ventricle was measured at 48 hpf to determine compound toxicity. Counting and recording of atrial and ventricular contraction were performed for 3 min using stereomicroscopy, and results were represented as the average heart rate per min [18].

### 2.15. MTT Assay for Cell Viability

Cell viabilities were assessed using the MTT assay, as previously described [20]. B16-F10 melanocytes were seeded into a 96-well plate at the density of 5 × 10^3^ cells/well for 12 hours. Cells were treated with compound or extract for 48 hr.

### 2.16. Statistical Analysis

Data were evaluated statistically using Student's *t*-test. Statistical significance was set at *P* < 0.05. The data are shown as the mean ± SEM of three independent experiments.

## 3. Results

### 3.1. Screening of* A. capillaris* Extract for Pigmentation Regulatory Activity Using a Melanocyte-Based Screening System

**Figure 1:**
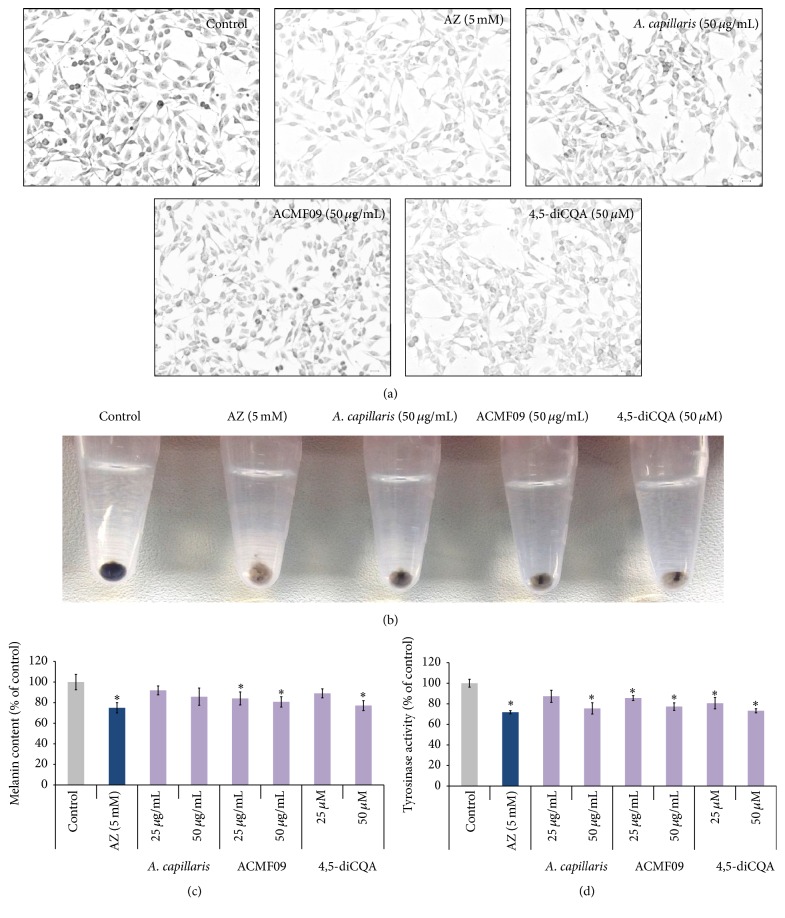
Inhibitory effect of the* A. capillaris* extract, active fraction ACMF09, and 4,5-diCQA on melanogenesis in B16-F10 melanocytes. (a) Phase-contrast microscopy of melanocytes showing less pigmented cells after exposure to test samples for 48 hr. Scale bar = 20 *µ*m. (b) Gross appearance of the cell pellets from treated melanocytes. (c) Melanin content in B16-F10 melanocytes treated with the* A. capillaris* extract, active fraction ACMF09, and 4,5-diCQA. (d) Tyrosinase activity in melanocytes after treatment with the indicated concentrations of test samples. Azelaic acid (AZ) was used as a positive control. The results are expressed as percentages of the untreated control, and the data are mean ± SEM of three independent experiments. ^*∗*^ *P* < 0.05 compared to the untreated control.

A methanol extract of the aerial parts of* A. capillaris* was screened to investigate its potential melanogenesis regulatory activity using murine melanocytes. Melanocytes were treated with two different concentrations: 25 and 50 *µ*g/mL of the extract for 48 hr. When B16-F10 melanocytes were treated with* A. capillaris*, the cells became visibly less dark compared to untreated cells, indicating reduced cellular melanogenesis (Figure 1(a)). This was confirmed by observation of the lightly colored crude extract of the treated cell pellet (Figure 1(b)). Treatment with 50 or 25 *µ*g/mL* A. capillaris* reduced melanin production to 91.92 ± 8.88% and 85.81 ± 10.12% compared to untreated melanocytes (Figure 1(c)). To assess the effect of* A. capillaris* on melanin content and tyrosinase activity, azelaic acid (AZ), a known tyrosinase inhibitor, was used as a positive control. Although previous studies reported that azelaic acid was effective at concentrations of 40 and 20 mM [21], we observed that treatment with these concentrations for 48 hr caused immediate detachment of more than 50% of the B16-F10 melanocytes (data not shown). We observed that 5 mM AZ treatment decreased melanin content by 15–20% without producing noticeable toxicity in the melanocytes (Figures 1(a)–1(c)). Subsequently, tyrosinase inhibition was assessed and it was observed that tyrosinase activity was partially inhibited by treatment with 25 or 50 *μ*g/mL of the* A. capillaris* extract for 48 h: 87.82 ± 0.065% and 75.68 ± 0.68% compared to untreated, respectively (Figure 1(d)).

### 3.2. Isolation and Characterization of an Antimelanogenesis Compound from the* A. capillaris* Extract

**Figure 2:**
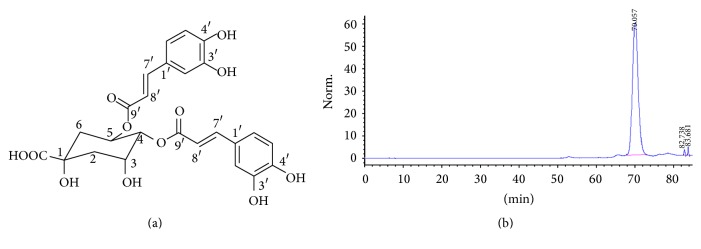
HPLC chromatogram of purified compound 4,5-diCQA. (a) Chemical structure of 4,5-diCQA. (b) 4,5-diCQA was isolated from* A. capillaris* by HPLC as described in Section 2.

High performance liquid chromatography (HPLC) was performed to isolate antimelanogenesis compounds (the chromatogram is shown in Supplementary Figure S1 in Supplementary Material available online at http://dx.doi.org/10.1155/2016/7823541). 23 fractions of varying weight were isolated from the* A. capillaris* plant extract via activity-guided separation (the fraction weights are shown in Supplementary Table 1). The fractions were collected, dried, and dissolved in DMSO and evaluated for their antimelanogenic activity at the same concentration used for the plant extract (25 *µ*g/mL). Four fractions (ACMF09, ACMF13, ACMF14, and ACMF23) were active and their antipigmentation activity was confirmed* in vivo *using zebrafish embryos (Supplementary Figure S2). Fraction ACMF09 was found to produce the greatest antimelanogenesis effect and selected for analysis in mammalian melanocytes. ACMF09 reduced pigmentation in the melanocytes (Figures 1(a) and 1(b)). Moreover, this fraction also significantly reduced melanin production and tyrosinase activity (Figures 1(c) and 1(d)). To isolate potential melanogenic regulatory compound(s), the ACMF09 was fractionated on a silica gel column and a reversed-phase HPLC column. A linear gradient solvent condition applied to HPLC separation was H_2_O-methanol and started at 60 : 40 (v : v) and kept constant for 50 min. The gradient system was then decreased to 0 : 100 and kept constant for 20 min. The mobile phase was delivered at the flow rate of 6.0 mL/min and detection of the eluate was carried out at 280 nm. We isolated and purified a compound identified as 4,5-*O*-dicaffeoylquinic acid (4,5-diCQA) on the basis of ^1^H-NMR and ESI-MS detector analysis (Figures 2(a) and 2(b)). This compound was a yellow amorphous powder. One spot was detected under UV at 280 nm. ^1^H- and ^13^C-NMR spectra of compound showed the existence of two caffeoyl moieties; six aromatic protons [*δ*_H_ 7.02 (1H, d, *J* = 1.8 Hz, H-2′ or H-2′′), 7.00 (1H, d, *J* = 1.8 Hz, H-2′ or H-2′′), 6.92 (1H, dd, *J* = 8.1, 1.8 Hz, H-6′ or H-6′′), 6.90 (1H, dd, *J* = 8.1, 1.8 Hz, H-6′ or H-6′′), 6.75 (1H, d, *J* = 8.1 Hz, H-5′ or H-5′′), 6.74 (1H, d, *J* = 8.1 Hz, H-5′ or H-5′′)] and trans doublets [*δ*_H_ 7.59 (1H, d, *J* = 15.9 Hz, H-7′ or H-7′′), 7.51 (1H, d,*J* = 15.9 Hz, H-7′ or H-7′′), 6.28 (1H, d, *J* = 15.9 Hz, H-8′ or H-8′′), 6.19 (1H, d, *J* = 15.9 Hz, H-8′ or H-8′′)], and two carboxyl groups (*δ*_C_ 168.7 and 168.4) and three hydroxyl carbons (*δ*_C_ 149.8, 146.9, and 146.8). ^1^H- and ^13^C-NMR spectra of the compound also showed the existence of a quinic acid moiety [*δ*_H_ 5.62 (1H, br s, H-5), 5.11 (1H, br s, H-4), 4.37 (1H, br s, H-3), 2.40 (2H, H-6), 1.99 (2H, m, H-2)]. The LC/UV/MS profile of the compound displayed UV absorption bands at 328, 292, and 245 nm and ESI-MS [M–H]^−^ peak at* m/z* 515.11. On the basis of these results, the structure of compound was elucidated as 4,5-*O*-dicaffeoylquinic acid [22]. 4,5-diCQA was examined for its effects on pigmentation in mammalian melanocytes. Microscopic inspection indicated that melanocyte pigmentation decreased after treatment with 4,5-diCQA (Figure 1(a)). The cell pellet was markedly lighter in color compared to control cells (Figure 1(b)). Quantitative analysis confirmed that 4,5-diCQA treatment reduced melanin level in the melanocytes (Figure 1(c)). In addition, 4,5-diCQA reduced tyrosinase activity in the melanocytes at the 50 *µ*M treatment concentration (Figure 1(d)).

### 3.3. Effects of Fraction ACMF09 and 4,5-diCQA on Mushroom Tyrosinase Activity in a Cell-Free System

**Figure 3:**
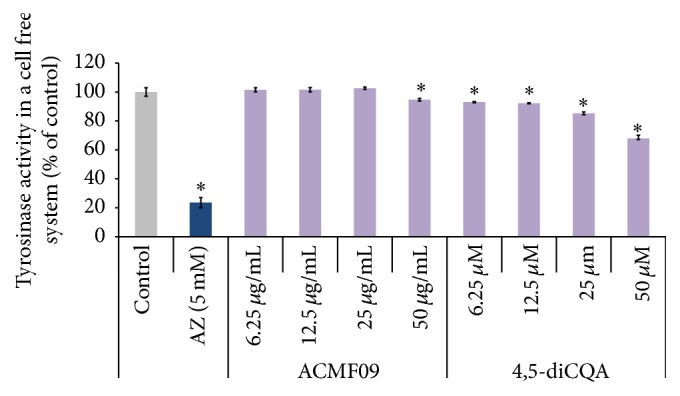
Inhibitory effect of the active fraction ACMF09 and 4,5-diCQA on tyrosinase activity. The inhibition of cell-free tyrosinase activity was determined using mushroom tyrosinase. Azelaic acid (AZ) was used as a positive control. The results are expressed as percentages of the control, and the data are mean ± SEM of three independent experiments. ^*∗*^ *P* < 0.05 as compared to the untreated control.

To investigate whether 4,5-diCQA directly inhibits the enzymatic activity of tyrosinase, the* in vitro* cell-free mushroom tyrosinase assay was used. It was observed that 4,5-diCQA inhibited mushroom tyrosinase in dose-dependent manner (Figure 3). At the 50 *µ*M concentration, 4,5-diCQA produced 32% enzyme inhibition compared to untreated. The positive control (5 mM AZ) produced a 77% inhibition of enzyme activity.

### 3.4. Effect of 4,5-diCQA on the Expression of MITF, Tyrosinase, and TRP-1

**Figure 4:**
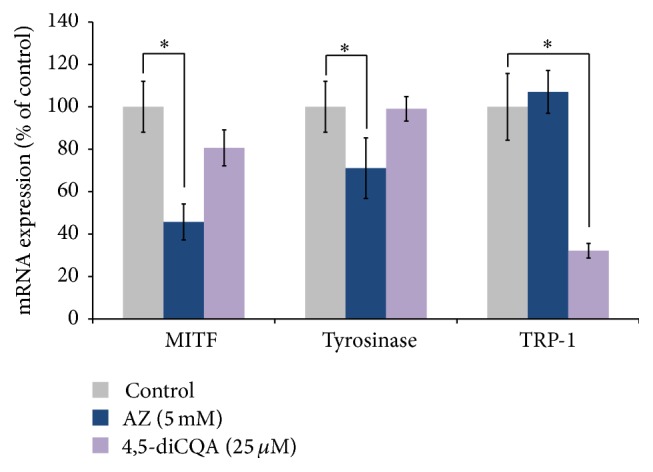
Quantitative real-time PCR analysis of the effect of 4,5-diCQA on the expression of the melanogenesis-related genes MITF, tyrosinase, and TRP-1 in B16-F10 melanocytes. mRNA signal was normalized to actin mRNA expression. Azelaic acid (AZ) was used as a positive control. The results are expressed as percentages of the control, and the data are mean ± SEM of three independent experiments. ^*∗*^ *P* < 0.05 as compared to the untreated control.

To explore the possible mechanism of the antipigmentation effects of 4,5-diCQA, the expression of levels of three key regulatory genes for melanogenesis, microphthalmia-associated transcription factor (MITF), tyrosinase-related protein-1 (TRP-1), and tyrosinase, was examined using quantitative real-time PCR. As shown in Figure 4, the mRNA level of tyrosinase was unchanged by treatment with 4,5-diCQA. In contrast, the mRNA expression of TRP-1 was significantly reduced.

### 3.5. *A. capillaris* Extract, ACMF09, and 4,5-diCQA Inhibit Pigmentation in the Zebrafish Vertebrate Model System

Zebrafish is an emerging animal model in pigmentation research [23]. We tested the* A. capillaries *methanol extract and ACMF09 active fraction in the zebrafish larvae-based* in vivo* system for comparison with the* in vitro* data. A well-known pigmentation inhibitor, 1-phenyl 2-thiourea (PTU), which reduces tyrosinase activity, was used as a positive control [24–26]. However, it has been reported that at the 28-somite stage, PTU may cause delayed hatching and mortality by 120 hpf [17]. Thus, for this study we tested different concentrations of PTU (25, 50, 75, 100, and 200 *µ*M) on zebrafish pigmentation. It was observed that the 75 *µ*M dose of PTU reduced pigmentation in the zebrafish, without significantly affecting mortality or producing teratogenic effects (data not shown).

**Figure 5:**
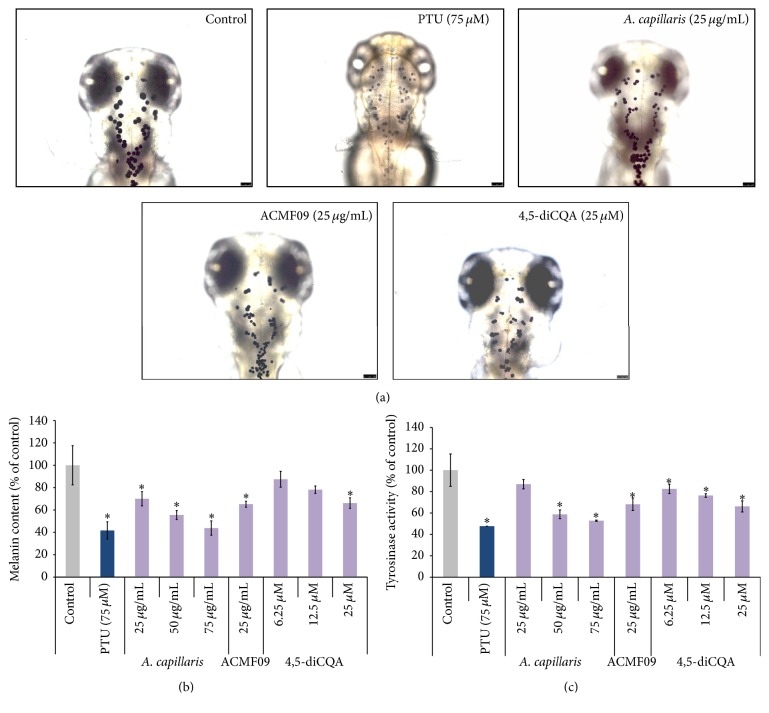
Inhibitory effect of the* A. capillaris* methanol extract, active fraction ACMF09, and 4,5-diCQA on pigmentation in developing zebrafish embryos. (a) Zebrafish was treated with test samples from 9 hfp to 72 hpf. Treatment with test samples at the indicated concentrations resulted in decreased pigmentation, as indicated by imaging the dorsal view of live embryos and the head portion. Scale bar = 250 *µ*m. (b) Melanin content in zebrafish embryos treated with test samples from 9 hfp to 48 hpf. (c) Tyrosinase activity in the treated zebrafish. PTU was used as positive control. Results are expressed as percentages of the control, and the data are mean ± SEM of three independent experiments. ^*∗*^ *P* < 0.05 compared to the untreated control.

The methanol extract of* A. capillaris*, fraction ACMF09, and 4,5-diCQA inhibited pigmentation in the zebrafish (Figure 5(a)). Interestingly, depigmentation was observed to be caused by shrinkage of the melanocytes in the head region of the embryos (Figure 5(a)). In addition, it was observed that the* A. capillaris* extract, fraction ACMF09, and 4,5-diCQA decreased melanin synthesis in zebrafish embryos in a dose-dependent manner (Figure 5(b)). Moreover, partial inhibition of tyrosinase activity was observed (Figure 5(c)), indicating that the reduction of melanogenesis in zebrafish is due to the partial inhibition of cellular tyrosinase activity.

### 3.6. Effect of 4,5-diCQA on Melanocyte Survival in the Zebrafish Larval Head Portion

**Figure 6:**
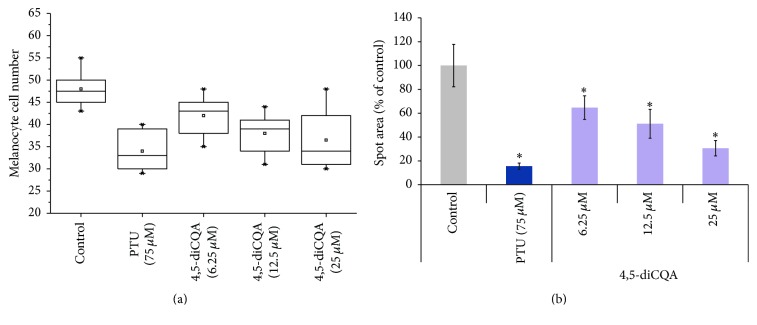
The effect of 4,5-diCQA on melanocyte cell number and spot area in zebrafish embryos. (a) Box and whisker plots of melanocytes number in the head region of 4,5-diCQA-treated embryos indicated no major difference compared with untreated embryos. The standard mean is indicated; outliers are represented by an asterisk. (b) Surface area of the melanocytes in zebrafish embryos. PTU was used as positive control. Azelaic acid (AZ) was used as a positive control. The results are expressed as percentages of control, and the data are the means ± SEM of three independent experiments. ^*∗*^ *P* < 0.05 as compared to the untreated control.

Melanocytes in zebrafish embryo begin to produce melanin at around 24 hpf. By 60 hpf there are approximately 460 melanocytes in the head, body, tail, and yolk sac that form the pattern of pigmentation [27]. In our study, we imaged melanocytes in the head region of the whole embryo. When 9 hpf larvae were incubated with 25 *µ*M 4,5-diCQA, the number of melanocytes was slightly less than in untreated the embryos (Figure 6(a)). 4,5-diCQA did not affect melanocyte cell number at the 12.5 *µ*M dose. However, melanocyte area was significantly decreased at all tested doses of 4,5-diCQA. For example, the 25 *µ*M dose reduced melanocyte area by approximately 4 times compared to untreated embryos (Figure 6(b)).

### 3.7. Determination of the Toxic Effects of* A. capillaris* Extract, ACMF09, and 4,5-diCQA* In Vitro* and* In Vivo*

**Figure 7:**
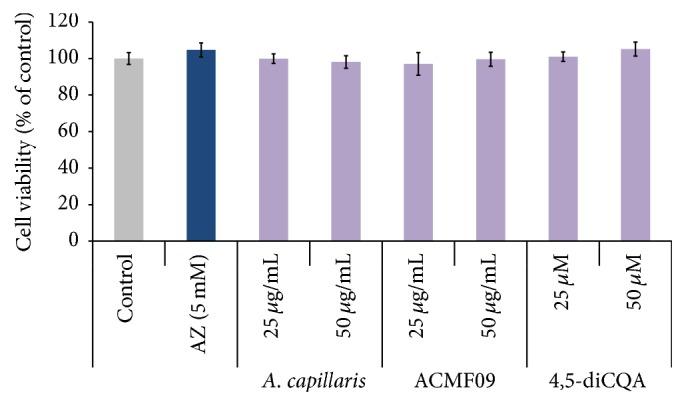
Effect of the* A. capillaris* extract, active fraction ACMF09, and 4,5-diCQA on melanocyte cell viability. B16-F10 melanocytes were treated with test samples at the indicated concentrations for 48 h. Cell viability was determined using the MTT assay. Azelaic acid (AZ) was used as a positive control. The results are expressed as percentages of the control, and the data are mean ± SEM of three independent experiments.

To confirm the effect of 4,5-diCQA on melanogenesis, the cytotoxicity of different concentrations of 4,5-diCQA on B16-F10 melanocytes was evaluated by MTT assay. As shown in Figure 7 cell viability did not change in the presence of 4,5-diCQA in all treatment groups compared to the control, indicating that the isolated compound is not cytotoxic to B16-F10 melanocytes. A very useful feature of zebrafish-based analysis is that the toxicity of candidate drugs can be readily tested in the developing larvae [28]. To evaluate the* A. capillaris* extract, ACMF09, and 4,5-diCQA for their potential toxic effects in zebrafish, we observed treated embryos at 24 hpf, 48, and 72 hpf for any morphological malformations, embryonic mortality, and heartbeat disturbances. We did not observe any adverse effects on zebrafish morphology and physiology. The zebrafish heart at 72 hpf is identical to that of a human embryo at three weeks' gestation stage and can be used as a toxic test for compound/compounds [29]. We observed that the average heart rate of treated embryos was not significantly different compared to untreated embryos. We also noted that the observed heart rates of treated embryos were not significantly different compared to untreated embryos. We also noted that the observed heart rates of treated embryos were similar to heart rates reported in previous studies [30–32].

## 4. Discussion

There is a research and therapeutic need to develop compounds that effectively regulate melanin synthesis [14]. Commonly utilized pigmentation inhibitors, such as corticosteroids or tyrosinase inhibitors, are effective but may produce toxic side effects [33]. In recent years, there has been renewed research in developing depigmenting products from natural sources, because there is greater potential to avoid safety issues [34]. Interestingly, the utilization of natural products to treat pigmentation has a long history. In Ancient China, it was common practice to use herbs to produce hypopigmentation [35, 36]. In our study, we used cell-based screening to examine the effect of* A. capillaris* on melanogenesis. To our knowledge, no study has been published concerning the melanogenic regulatory activity of this plant. In our study, we have demonstrated that an* A. capillaris* extract produces pronounced inhibitory effects on pigmentation in B16-F10 melanocytes in a dose-dependent manner. Our data showed that fraction ACMF09 displayed the antityrosinase activity in the melanocytes and zebrafish system. We isolated the potential pigmentation inhibitor compound from ACMF09 using HPLC and identified it as 4,5-diCQA. This compound has been shown to have many pharmacological properties [37–41]. Our study is the first to show that 4,5-diCQA can be isolated from* A. capillaries* and can suppress melanin biosynthesis in B16-F10 melanocytes via partial inhibition of tyrosinase activity. 4,5-diCQA has also been extracted from other plant sources, for example, green coffee beans [42] and* Gnaphalium affine* D. DON [43]. These previous studies also showed that 4,5-diCQA inhibited tyrosinase activity and produced antioxidant effects. However, these previous studies only assessed 4,5-diCQA using enzyme-based, cell-free systems; there was no assessment on pigmentation in cells or animal models.

Our real-time PCR analysis of tyrosinase indicated that 4,5-diCQA does not affect gene expression. This is consistent with our finding that 4,5-diCQA partially inhibits tyrosinase enzyme activity. Moreover, our finding that 4,5-diCQA showed significant inhibitory effects on TRP-1 expression indicates that this compound may also inhibit pigmentation by targeting TRP-1 (TRP-1 has been demonstrated to activate the tyrosinase and enhance its stability and thus induce melanin synthesis [44]). Elucidating exactly how 4,5-diCQA downregulates both TRP-1 and its effect on pigmentation relative to tyrosinase enzyme inhibition could be an interesting avenue for further research.

The* in vivo* imaging data of zebrafish melanocytes indicated that 4,5-diCQA caused shrinkage of these cells. To our knowledge, melanocyte shrinkage without cytotoxicity is not a common feature of depigmentation compounds (our MTT data suggests that 4,5-diCQA is not cytotoxic for melanocytes, even though some studies demonstrate that phenolic compounds produce melanocyte toxicity) [27, 45]. It has been shown that cytochalasin B (a mycotoxin that inhibits actin filament formation) or dysregulation of melanocyte function by T-helper cell 17-related cytokines can induce melanocyte shrinkage [46, 47].

Our compound also caused a slight reduction in melanocyte numbers in the zebrafish skin. This decrease in melanocyte numbers could be due to factors such as melanocyte cell death, clustering of melanocytes to make them indistinguishable as separate units, or the inhibition of melanoblast proliferation [45]. Assessing the precise mechanism of 4,5-diCQA on melanocyte morphology* in vivo* should be an interesting area for future investigation. Many candidate drugs have been shown to induce toxic effects by targeting the circulatory system [48]. It has been demonstrated that the pharmacological responses of zebrafish to various classes of drugs, and the development of the cardiovascular system, are markedly similar to humans [49, 50]. Our results revealed that 4,5-diCQA produced antipigmentation effects* in vivo* with no obvious developmental defects or effect on heart rate.

In summary, in this work we employed melanocyte-based screening for the activity-guided fractionation of* Artemisia capillaris* Thunberg to identify pigmentation regulatory compounds. The cell-based screening was coupled with validation in the zebrafish animal model. Using this approach, we identified 4,5-diCQA as a depigmenting compound that inhibits tyrosinase activity and is effective* in vivo*. Our results further support the zebrafish as a valuable model for drug discovery, which has also been demonstrated in other disease contexts, such as diabetes and cancer [51, 52]. The discovery of 4,5-diCQA as a nontoxic cosmetic and pharmaceutical depigmenting should be of interest to companies developing skin whitening products, in addition to researchers studying melanogenesis.

## Supplementary Material

1) HPLC chromatogram of the stem and leaves of *Artemisia capillaris*, 2) Weights of the fractions extracted from *Artemisia capillaris*, and 3) Inhibitory effects of active fractions ACMF09, ACMF13, ACMF14, and ACMF23 on pigmentation in developing zebrafish embryos.

## Abbreviations

^13^C-NMR: Carbon 13 nuclear magnetic resonance
^1^H-NMR: Proton nuclear magnetic resonance
4,5-diCQA: 4,5-*O*-Dicaffeoylquinic acid
*A. capillaris*: * Artemisia capillaris*
ACMF09: * Artemisia capillaris* MeOH Fraction number 09
AZ: Azelaic acid
DMSO: Dimethyl sulfoxide
Hpf: Hours postfertilization
HPLC: High performance liquid chromatography
L-DOPA: L-3,4-Dihydroxyphenylalanine
MeCN: Acetonitrile
MeOH: Methanol
MITF: Microphthalmia-associated transcription factor
NaOH: Sodium hydroxide
PTU: 1-Phenyl 2-thiourea
TRP-1: Tyrosinase-related protein-1.
