# Supplementary material for: Isolation of 4,5-O-Dicaffeoylquinic Acid as a Pigmentation Inhibitor Occurring in Artemisia capillaris Thunberg and Its Validation In Vivo
Source: Evid Based Complement Alternat Med. 2016 Jul 26;2016:7823541. doi: 10.1155/2016/7823541 (PMC4977398; doi:10.1155/2016/7823541)
Supplement: Supplementary file 1 — 1) HPLC chromatogram of the stem and leaves of Artemisia capillaris, 2) Weights of the fractions extracted from Artemisia capillaris, and 3) Inhibitory effects of active fractions ACMF09, ACMF13, ACMF14, and ACMF23 on pigmentation in developing zebrafish embryos. [file 7823541.f1.docx]

**Isolation of 4,5-O-dicaffeoylquinic acid as a pigmentation inhibitor occurring in *Artemisia capillaries* Thunberg and its validation *in vivo***

**Nadia Tabassum^1^**^†^**, JiHyung Lee^1^**^†^**, Soon-Ho Yim^2^, Galzad Javzan Batkhuu^3^, Da-Woon Jung^1*^, Darren R. Williams^1*^**

*^1^New Drug Targets Laboratory, School of Life Sciences, Gwangju Institute of Science and Technology, Gwangju 500-712, Republic of Korea*

*^2^Department of Pharmaceutical Engineering, Dongshin Unviersity, Naju, Jeonnam, Republic of Korea*

*^3^School of Engineering and Applied Sciences, National University of Mongolia, Ulaabaatar, Mongolia*


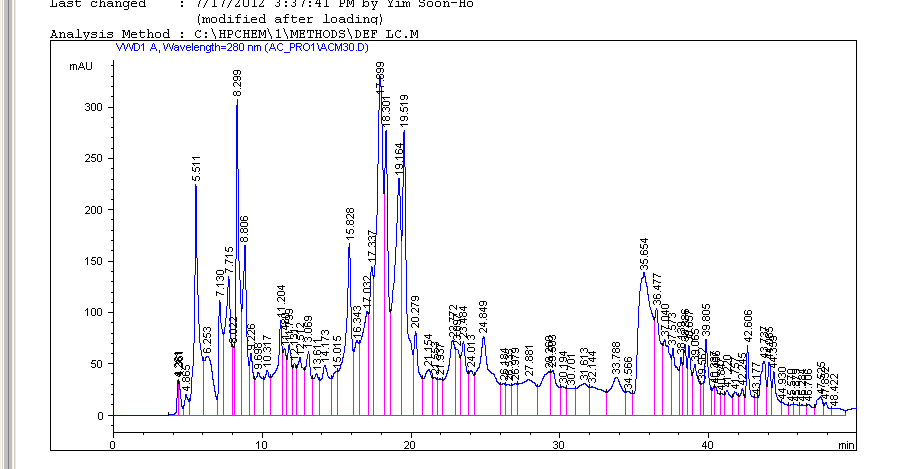


SUPPLEMENTARY FIGURE 1: High-performance liquid chromatography (HPLC) chromatogram of leaves and stems *Artemisia capillaris* with indication of active fraction ACMF09

SUPPLEMETARY FIGURE S1: High-performance liquid chromatography (HPLC) chromatogram of stem and leaves of *Artemisia capillaris* with indication of active fraction ACMF09.

SUPPLEMENTARY TABLE 1: Weight (mg) of the fractions extracted from *Artemisia capillaris*

| **S.N0** | **Fraction Number** | **Weight (mg)** |
| --- | --- | --- |
| **1** | ACMF01 | 266 |
| **2** | ACMF02 | 191 |
| **3** | ACMF03 | 450.1 |
| **4** | ACMF04 | 131 |
| **5** | ACMF05 | 236 |
| **6** | ACMF06 | 62.9 |
| **7** | ACMF07 | 101.3 |
| **8** | ACMF08 | 152.4 |
| **9** | ACMF09 | 69.3 |
| **10** | ACMF10 | 64.4 |
| **11** | ACMF11 | 168 |
| **12** | ACMF12 | 98 |
| **13** | ACMF13 | 118.9 |
| **14** | ACMF14 | 88.2 |
| **15** | ACMF15 | 104.9 |
| **16** | ACMF16 | 200 |
| **17** | ACMF17 | 212 |
| **18** | ACMF18 | 141.1 |
| **19** | ACMF19 | 190 |
| **20** | ACMF20 | 195 |
| **21** | ACMF21 | 172.1 |
| **22** | ACMF22 | 320 |
| **23** | ACMF23 | 131 |


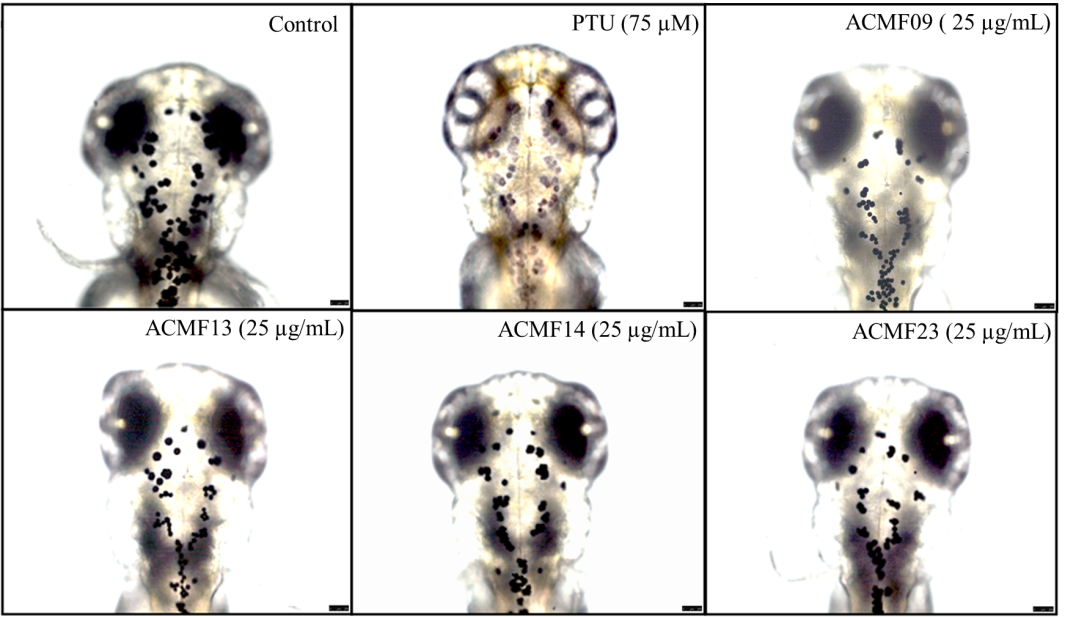


(a)


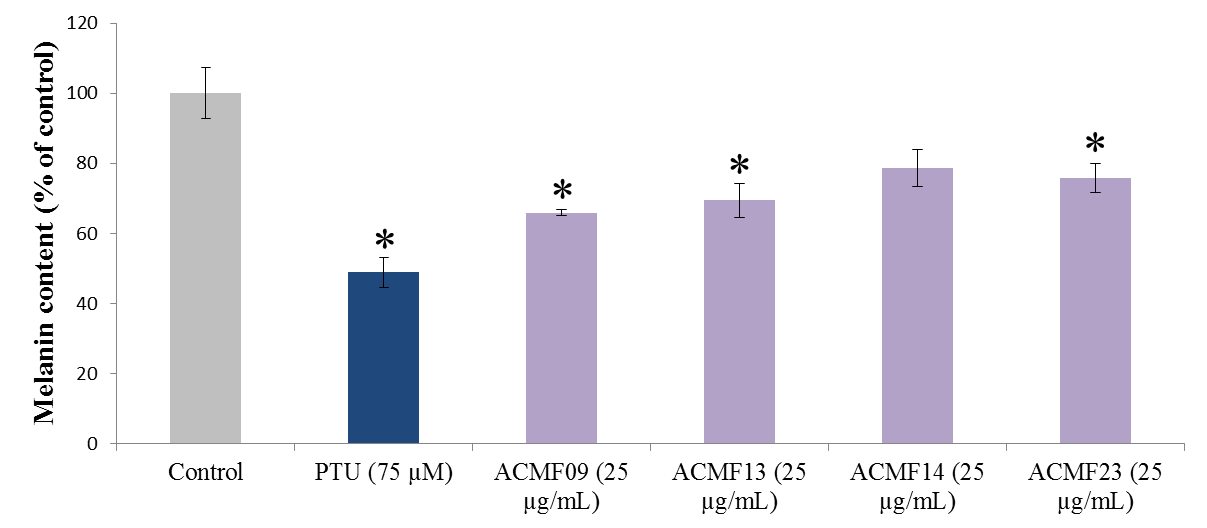


(b)


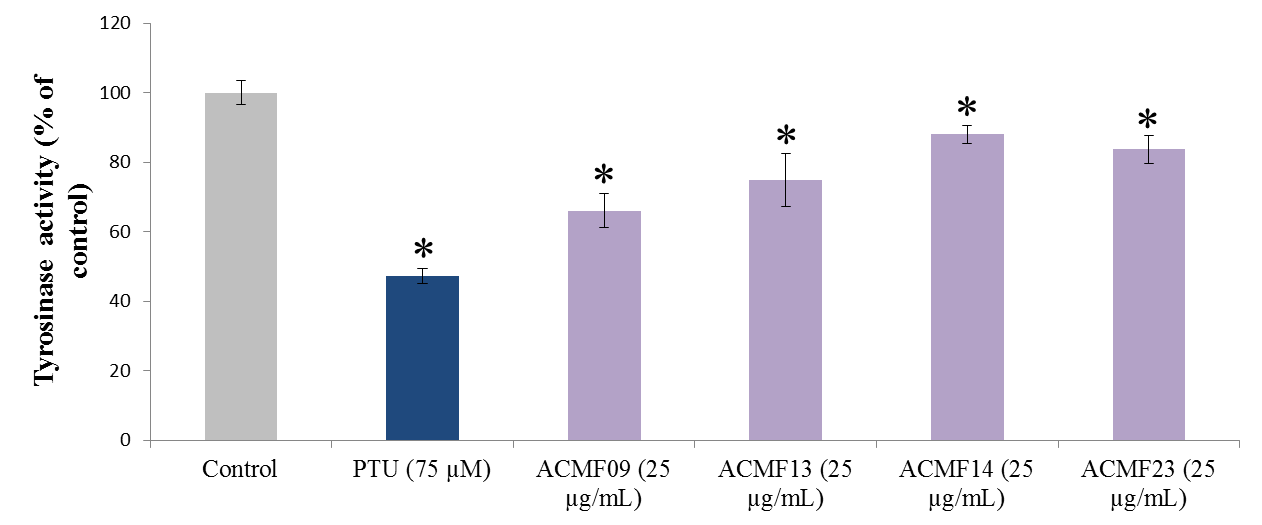


(c)

SUPPLEMENTARY FIGURE S2: Inhibitory effect active fractions ACMF09, ACMF13, ACMF14, and ACMF23 on pigmentation in developing zebrafish embryos. (a) Zebrafish were treated with test samples from 9 hfp to 72 hpf. Treatment with test samples at the indicated concentrations resulted in decreased pigmentation as indicated by imaging the dorsal view of live embryos and the head portion Scale bar= 250 µm. (b) Melanin content in zebrafish embryos treated with active fractions from 9 hfp to 48 hpf. (c) Tyrosinase activity in the treated zebrafish. PTU was used as positive control. Results are expressed as percentages of the control, and the data are mean ± SEM of three independent experiments. **p*<0.05 compared to the untreated control.
